# Biological basis and clinical study of glycogen synthase kinase- 3β-targeted therapy by drug repositioning for glioblastoma

**DOI:** 10.18632/oncotarget.15206

**Published:** 2017-02-09

**Authors:** Takuya Furuta, Hemragul Sabit, Yu Dong, Katsuyoshi Miyashita, Masashi Kinoshita, Naoyuki Uchiyama, Yasuhiko Hayashi, Yutaka Hayashi, Toshinari Minamoto, Mitsutoshi Nakada

**Affiliations:** ^1^ Department of Neurosurgery, Division of Neuroscience, Graduate School of Medical Science, Kanazawa University, Kanazawa, Japan; ^2^ Division of Translational and Clinical Oncology, Cancer Research Institute, Kanazawa University, Kanazawa, Japan

**Keywords:** glioblastoma, GSK3β, drug repositioning, translational research, clinical study

## Abstract

**Background:**

Glycogen synthase kinase (GSK)-3β has emerged as an appealing therapeutic target for glioblastoma (GBM). Here, we investigated the therapeutic effect of the current approved drugs against GBM via inhibition of GSK3β activity both, in experimental setting and in a clinical study for recurrent GBM patients by repositioning existent drugs in combination with temozolomide (TMZ).

**Materials and Methods:**

Progression-free and overall survival rates were compared between patients with low or high expression of active GSK3β in the primary tumor. GBM cells and a mouse model were examined for the effects of GSK3β-inhibitory drugs, cimetidine, lithium, olanzapine, and valproate. The safety and efficacy of the cocktail of these drugs (CLOVA cocktail) in combination with TMZ were tested in the mouse model and in a clinical study for recurrent GBM patients.

**Results:**

Activation of GSK3β in the tumor inversely correlated with patient survival as an independent prognostic factor. CLOVA cocktail significantly inhibited cell invasion and proliferation. The patients treated with CLOVA cocktail in combination with TMZ showed increased survival compared to the control group treated with TMZ alone.

**Conclusions:**

Repositioning of the GSK3β-inhibitory drugs improved the prognosis of refractory GBM patients with active GSK3β in tumors. Combination of CLOVA cocktail and TMZ is a promising approach for recurrent GBM.

## INTRODUCTION

Glioblastoma (GBM), one of the most common primary brain tumor in adults, is highly malignant with exhibiting aggressive clinical manifestation and a median life expectancy of less than 2 years [1]. Biologically, GBM is characterized by its high proliferative and invasive activity [2]. Only a few drugs were prescribed for GBM: temozolomide (TMZ) [3], biodegradable carmustine (BCNU) wafer [4, 5], and bevacizumab [6, 7]. Till date, no effective therapeutic for GBM has emerged despite the development of a variety of molecular-targeted agents such as tyrosine kinase inhibitors [8].

Glycogen synthase kinase (GSK)-3β is a serine/threonine protein kinase that regulates various cellular pathways depending on its substrates by phosphorylation [9]. Emerging evidence suggests that GSK3β promotes GBM progression by interacting with the pivotal molecules that participate in tumor cell survival, proliferation, invasion, and resistance to chemoradiation therapy [10, 11]. We and others have reported that GSK3β inhibition attenuates the proliferation, migration and invasion of GBM cells by modulating distinct molecular pathways, and sensitizes them to chemotherapeutic agents and radiation [12–19]. These studies have substantiated GSK3β as therapeutic target of GBM.

Despite experimental evidence supporting the therapeutic effect of GSK3β inhibition, none of GSK3β inhibitors was approved for treatment of diseases such as diabetes mellitus, Alzheimer's disease, and cancer. The development and approval of new drug is a time-consuming and expensive process with high rate of failure. Recently, drug repositioning to find new applications for existing drugs has emerged to overcome this challenge [20]. Drug repositioning attempts to discover new therapeutic options for already approved drugs, as their pharmacokinetics, pharmacodynamics and post-marketing surveillance safety data are readily available [21]. Drugs such as cimetidine, lithium carbonate, olanzapine, and valproate, prescribed for other diseases inhibited GSK3β activity [22–24]. In the present study, these GSK3β-inhibiting drugs were examined for therapeutic effect against GBM in experimental settings. We also explored whether GSK3β activity in clinical tumor tissues influences the survival of GBM patients to rationalize the targeting of GSK3β. Based on these experimental and clinical findings, we conducted a clinical study to investigate the safety and therapeutic effect of repositioning GSK3β-inhibiting medicines in combination with TMZ, in patients with recurrent GBM.

## RESULTS

### Activated GSK3β is an unfavorable prognostic marker in GBM

**Figure 1:**
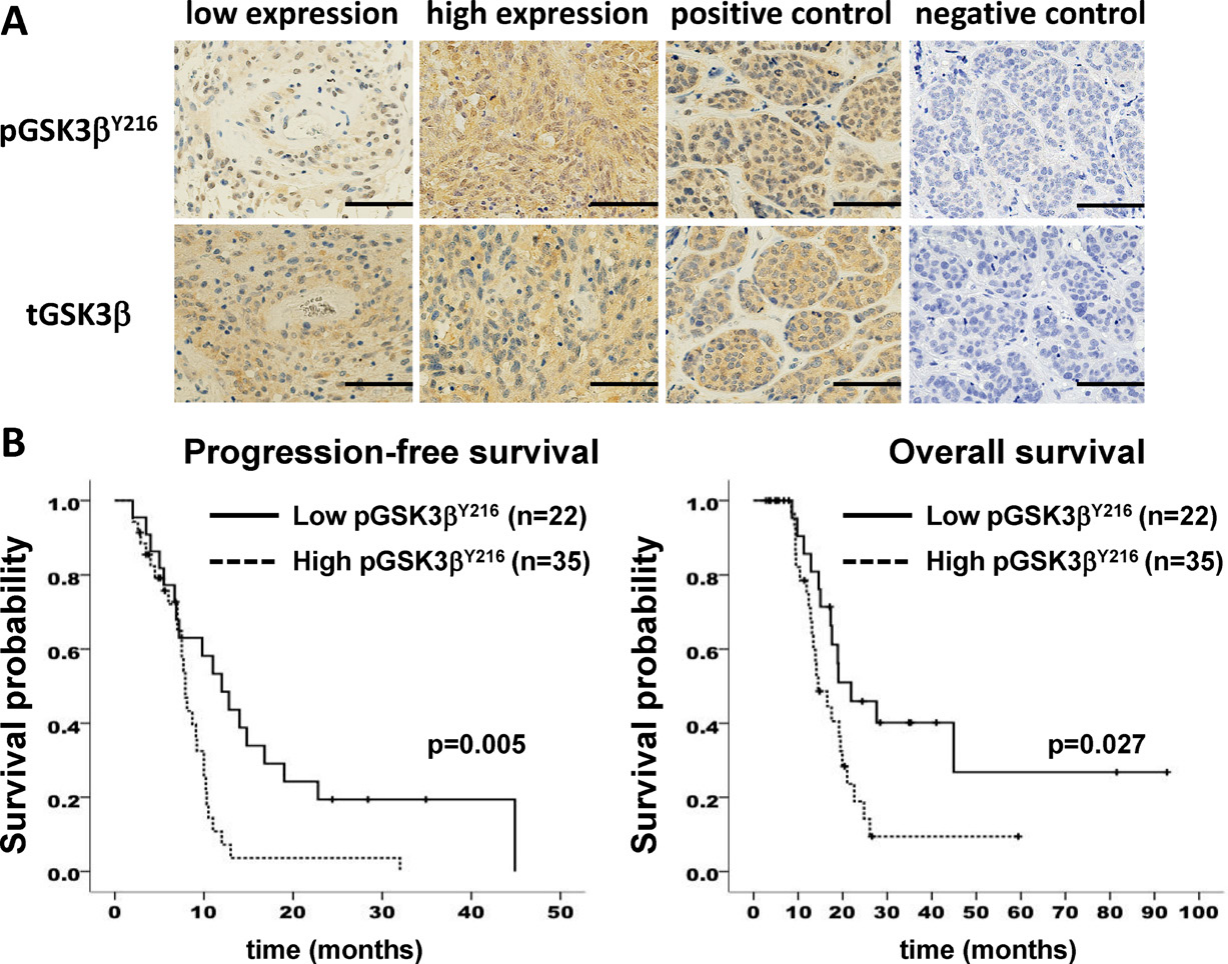
GSK3β activation correlates with poor survival in patients with glioblastoma (**A**) immunohistochemical analysis of human glioblastoma samples for pGSK3β^Y216^ and total (t) GSK3β. Positive and negative control was a case of metastatic breast carcinoma. *Scale bar*, 50 μm. (**B**) Kaplan-Meier curves showing survival of glioblastoma patients based on expression level of pGSK3β^Y216^. *n*, number of patients.

First, we investigated the expression of GSK3β and pGSK3β^Y216^ (active form) in clinical samples. Interestingly, survival analysis of 57 patients with GBM showed a significant correlation between pGSK3β^Y216^ expression and both progression-free survival (PFS) and overall survival (OS) (Figure 1). Subgroup analysis of age, Karnofsky Performance Scale (KPS), and O^6^-methylguanine-DNA-methyltransferase (MGMT) promoter status, and extent of resection (EOR) showed no statistical difference between GSK high group and GSK low group (Supplementary Table 1). Univariate analysis by Kaplan-Meier method showed methylated MGMT promoter, less age, and high EOR as predictive factors in our cohort (Supplementary Figure 1). Multivariate analysis revealed that activation of GSK3β was an independent unfavorable predictive factor (Table 1).

**Table 1:** Multivariate predictors of survival in our cohort

Variable	Progression-free survival			Overall survival		
	RR	95% CI	p*	RR	95% CI	p*
Low pGSK3β^Y216^	2.15	1.08–4.31	0.030	2.41	1.12–5.25	0.026
Age < 65	1.66	0.81–3.41	0.166	1.90	0.78–4.60	0.155
KPS ≥ 80%	1.49	0.76–2.92	0.247	1.06	0.47 –2.41	0.890
Methylated MGMT promoter	2.30	1.17–4.52	0.015	0.99	0.49–2.01	0.977
EOR ≥ 80%	1.63	0.76–3.51	0.212	1.94	0.79–4.78	0.151

Abbreviations: CI, confidence interval; EOR, extent of resection; GSK3β, glycogen synthase kinase 3β; KPS, Karnofsky Performance Scale; MGMT, O^6^-methylguranine-DNA-methyltransferase; pGSK3β^Y216^, GSK3β phosphorylated at tyrosine (Y) 216 residue; RR, relative risk.

*Cox regression model.

### Inhibition of GSK3β activity by existing drugs

**Figure 2:**
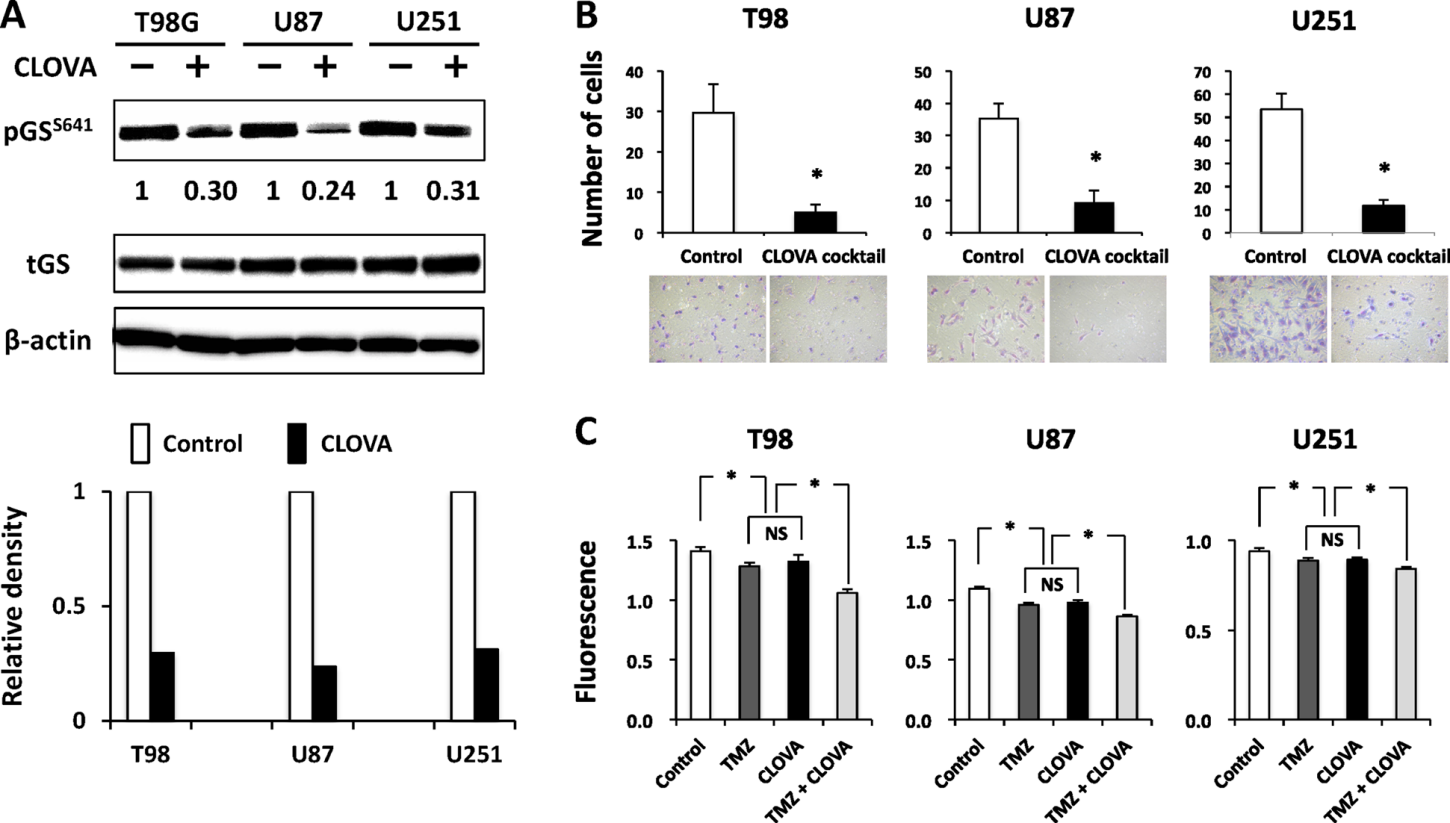
Effect of treatment with the CLOVA cocktail on the level of serine 641 phosphorylation of glycogen synthase (pGS^S641^) Expression level of pGS^S641^ (**A**), invasion (**B**) and proliferation (**C**) in GBM cells. Cells were treated with the CLOVA cocktail (the mixture of cimetidine, 0.1 mM; lithium, 1 mM; olanzapine, 0.1 μM; and valproate, 1 mM). A, Western blotting analysis for changes in the levels of pGS^S641^ in GBM cells following treatment with the CLOVA cocktail for 24 hours. The value below the blots shows relative level of pGS^S641^ quantified by densitometry and normalized to that of total (t) GS. All blots were cropped for clarity and conciseness of the images. B, Invading cells through a Matrigel-coated Transwell chamber were scored for cells treated with or without CLOVA cocktail for 8 hours. The mean number of cells in eight high-power microscopic fields was calculated with standard deviations (SDs). C, Growth analysis of GBM cell lines treated with the indicated reagents or without treatment (Control) for 96 h. The plate was read on fluorescence plate reader. B and C: NS, not significant; *p < 0.05.

We examined whether the existent drugs (cimetidine, lithium, olanzapine and valproate) inhibit GSK3β activity in cells by monitoring the level of pGS^S641^, the primary substrate of GSK3β (reviewed in ref. [9]). The levels of pGS^S641^ in all GBM cells decreased following treatment with each drug (Supplementary Figure 2). Hereafter the combination of these drugs is referred as “CLOVA cocktail” by the initial letters of each drug. While there were some differences in the GSK3β-inhibitory effect among the drugs and cell lines, the CLOVA cocktail decreased the level of pGS^S641^ in all GBM cells more efficiently than each of the cocktail constituents (Figure 2A). The results indicate that doses of these four medicines mixed in the CLOVA cocktail are within the ranges of clinical use nevertheless this cocktail inhibits GSK3β activity in GBM cells.

### Effects of the GSK3β-inhibiting drugs on invasion and proliferation of GBM cells

The Transwell assay showed that all 4 drugs inhibited the invasion of GBM cells to extracellular matrix (Supplementary Figure 3A). Lithium showed proliferation-inhibition at 5 mM and 10 mM in T98 and U87, while only at 10 mM in U251 cells. Valproate at 5 mM and 10 mM inhibited proliferation of all the 3 cell lines. However, neither cimetidine nor olanzapine inhibited cell proliferation at the indicated concentrations (Supplementary Figure 3B), and this issue was discussed later.

Next, we investigated the effect of CLOVA cocktail that consists of the 4 drugs as a mixture at the lowest concentrations (cimetidine, 0.1 mM; lithium, 1 mM; olanzapine, 0.1 μM; and valproate, 1 mM) concomitant with TMZ on GBM invasion and proliferation. The concentrations of cimetidine, lithium, and valproate used in culture medium were determined according to their blood concentration at the administration of maximum daily dose, because the blood-brain barrier (BBB) is damaged in GBM tissue. The dose of olanzapine was calculated based on its intracerebral concentration at the administration of maximum daily dose and its high BBB permeability and accumulation in brain tissue [25]. As expected, CLOVA cocktail itself showed additive inhibitory effect on cell invasion (Figure 2B) while TMZ showed no effect at the same time point (data not shown). CLOVA cocktail and TMZ inhibited cell proliferation equally, and moreover the combination of them showed remarkable inhibitory effect on cell proliferation (Figure 2C). The results shown in Figure 2, Supplementary Figures 2 and 3 collectively suggest a causal association between the effects of CLOVA cocktail against the GSK3β activity of GBM cells and their proliferative and invasive capacity.

### Effect of CLOVA cocktail on GBM cell invasion and proliferation in a brain tumor model

**Figure 3:**
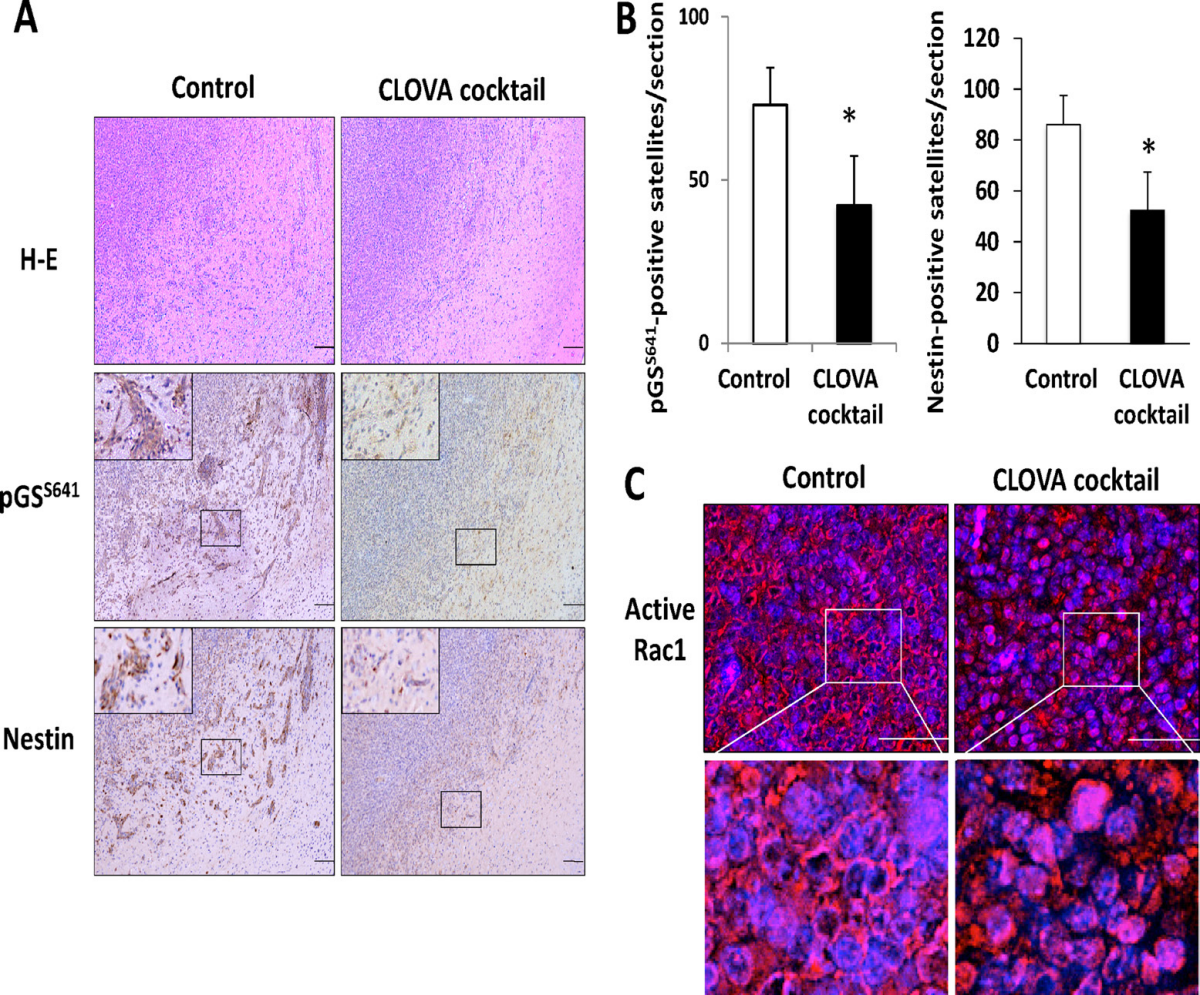
Effect of CLOVA cocktail on the glioblastoma animal model (**A**) Representative histological and immunohistochemical sections of brain tumors treated with or without CLOVA cocktail. Both activity of GSK3β (estimated by the level of pGS^S641^) and expression of nestin decreased in the satellite lesions. Mice treated with CLOVA cocktail showed a well-demarcated border between the tumor and adjacent normal brain tissue. The magnified image of the area in the square is shown in the left upper corner in each panel of pGS^S641^ and nestin. *Scale bars*, 100 μm. (**B**) Effects of CLOVA cocktail on the number of pGS^S641^- and nestin-positive cell clusters. *p < 0.05. (**C**) Immunofluorescence microscopic findings of tumor tissues for active Rac1 (red). Cell nuclei were counterstained with Hoechst 33342 (blue). Subcellular localization of active Rac1 changed from cellular rim to whole cytoplasm following treatment with CLOVA cocktail. Magnified images of the area in the square are shown in the lower panels. pGS^S641^, glycogen synthase (GS) phosphorylated at serine 641 residue.

The tumor histology of our animal model demonstrated several features, characteristic of human GBM, including high proliferative and invasive nature. Inhibition of GSK3β activity by treatment with each drug and the CLOVA cocktail was confirmed by the decreased level of pGS^S641^ in tumor cells, especially in satellite lesions, and the effect of CLOVA cocktail was strongest (Figure 3A, Supplementary Figure 4). Similarly, the number of diffusely infiltrating tumor cells that stained positive for nestin significantly decreased in CLOVA cocktail-treated mice (Figure 3A and 3B). Consequently, well-demarcated border between the tumor and adjacent normal brain tissues was observed both in mice treated with each drug alone and the combination (Supplementary Figure 4, Figure 3). Previous studies showed that focal adhesion kinase (FAK) and Rac1 interact [26] and facilitate GBM invasion [27, 28]. The attenuated invasive capacity of GBM tumors following treatment with CLOVA cocktail was associated with decrease in activating phosphorylation of (pFAK^Y397^ and pFAK^Y861^; Supplementary Figure 5) and with alteration in subcellular localization of active Rac1 in tumor cells (Figure 3C). These findings are consistent with our earlier study showing that GSK3β inhibition decreased active Rac1 fraction and FAK phosphorylation in human GBM cell lines [29].

**Figure 4:**
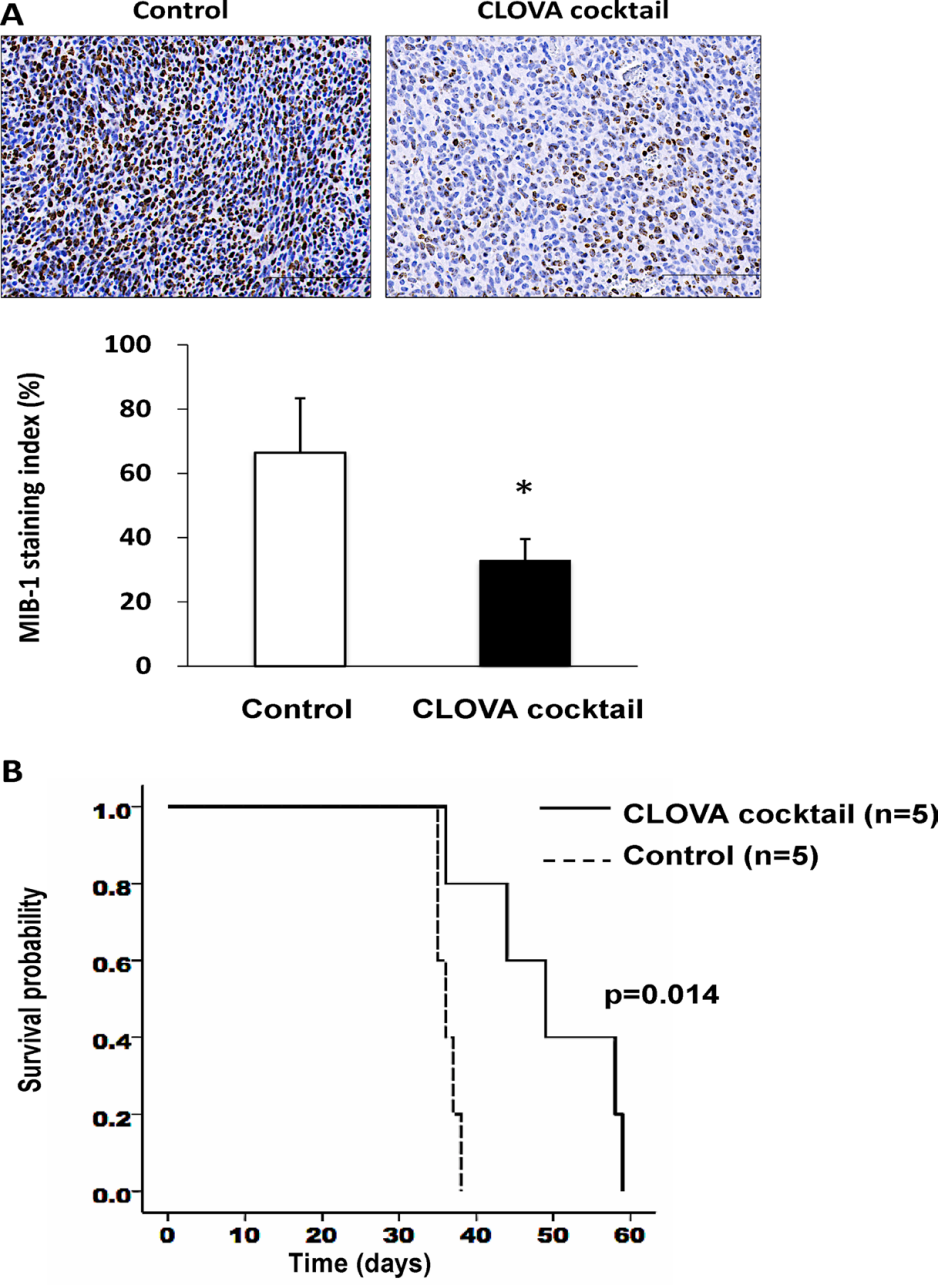
Effect of CLOVA cocktail on the glioblastoma animal model (**A**) Immunohistochemistry of MIB-1 and the staining index (%) indicating activity of cell proliferation. *Scale bars*, 100 μm. *p < 0.05. (**B**) Survival of mice treated with CLOVA cocktail and those treated with DMSO/H_2_O (Control). Log-rank test, *p* = 0.014. *n*, number of mice.

To compare the tumor proliferative potential, MIB-1 immunostaining was performed. Interestingly, MIB-1 staining index remarkably decreased in CLOVA cocktail treated-mice (Figure 4A), while less marked reduction was observed in mice receiving individual drug treatment (Supplementary Figure 4). TMZ alone and in combination with the CLOVA cocktail reduced the tumor size while the effects were not statistically significant (Supplementary Figure 6). Of note, CLOVA cocktail significantly prolonged survival of the mouse model (Figure 4B).

### Phase I/II clinical study

**Figure 5:**
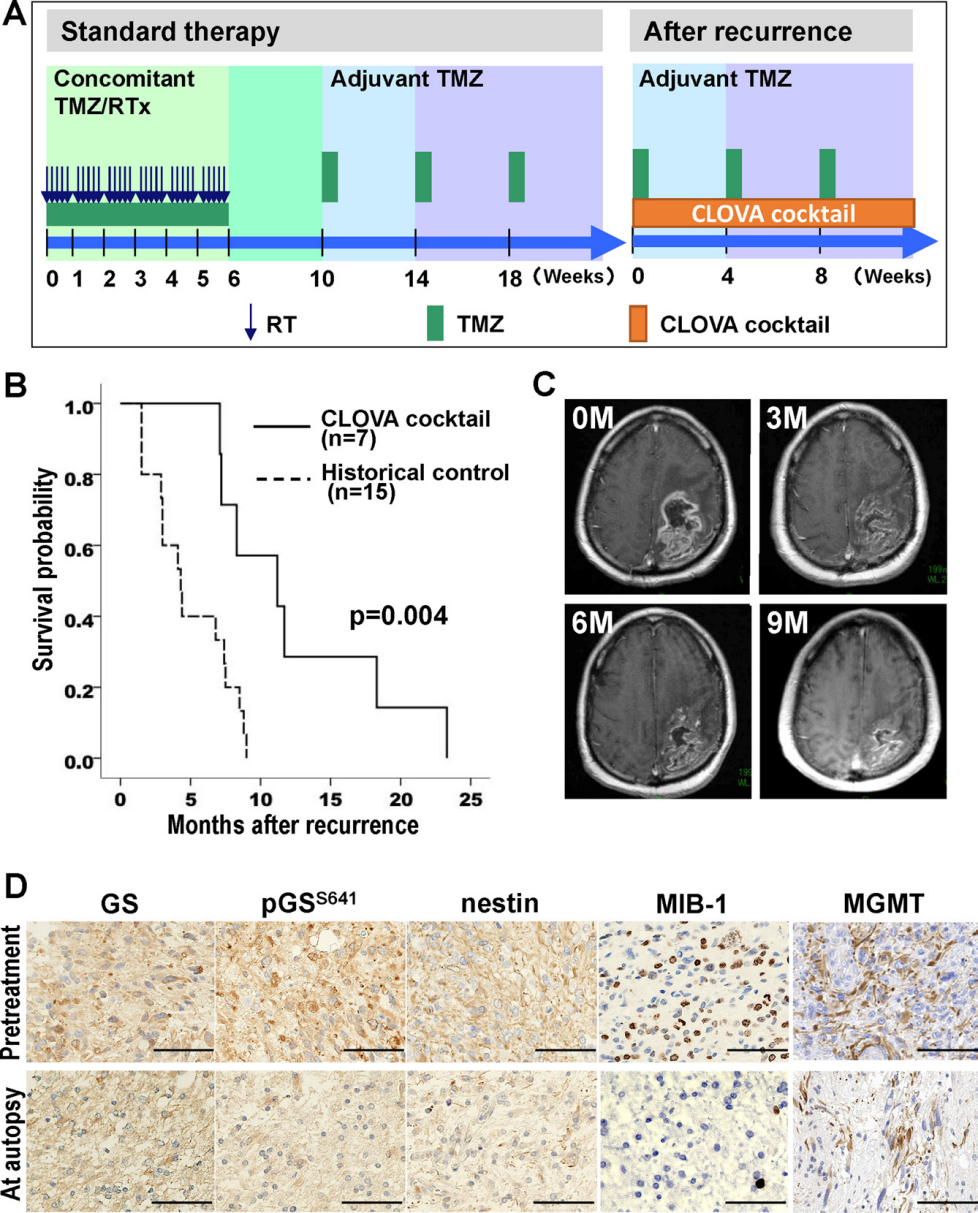
Phase I/II clinical study (**A**) Treatment protocol in the clinical study. (**B**) Overall survival of the patients treated with or without CLOVA cocktail in combination with temozolomide. (**C**) MRI images of a representative case (Case No. 6 in Table 2) of 77 y.o. male with recurrent GBM of the left parietal lobe. Three (3M), six (6M), and nine months (9M) after beginning of CLOVA cocktail (0M), the recurrent tumor adjacent to the resection cavity shrank continuously. All MRI images were post-contrast axial T1-weighted. (**D**) Immunohistochemical findings of the recurrent tumor before the treatment and the tumor obtained by autopsy in the case shown in C. *Scale bar* 50 μm; GS, glycogen synthase; pGS^S641^, GS phosphorylated at serine 641 residue; MGMT, O^6^-methylguanine-DNA methyltransferase; RTx, radiotherapy; TMZ, temozolomide.

Seven patients were enrolled in this study (Figure 5A). All patients were eligible following radiological disease progression (Macdonald criteria [30]) and classified as Recursive Partitioning Analysis (RPA) class 7 [31]. Median age was 66 years and median KPS was 50. MGMT promoter methylation was determined in 4 patients (57%). Three patients took CLOVA cocktail on the day before death and 4 patients discontinued the oral medication because of aspiration pneumonitis, 2 weeks before death. Median OS after first recurrence was 11.2 (95% CI, 3.8–18.6) months in patients treated with concomitant CLOVA cocktail with maintenance TMZ although they were classified into RPA class 7 wherein the median survival duration was estimated as 4.9 months [31], compared to median OS of 4.3 (95% CI, 2.5–6.1) months in historical control group (log-rank test, *p* = 0.004) (Figure 5B). The concomitant CLOVA cocktail with TMZ was safe and well tolerated. Most common treatment-related AE was grade 1/2 somnolence caused by antipsychotic drug constituents of CLOVA cocktail (Table 2). A representative case showed marked reduction of the recurrent tumor assessed as partial response (PR) 9 months after beginning of CLOVA cocktail (Figure 5C). Immunohistochemistry using the respective antibodies (Supplementary Table 2) of pretreatment and autopsy tissue showed decrease in pGS^S641^, nestin, MIB-1 index (pretreatment 32.9% to autopsy 8.0%) and MGMT levels (Figure 5D), suggesting that the CLOVA cocktail treatment inhibited GSK3β activity, attenuated tumor invasion and enhanced TMZ effect, similar to *in vivo* experiment (Figures 3 and 4).

**Table 2:** Patient characteristic enrolled and the results of the clinical study

Case	Age	Sex	KPS	MGMT*	Response†	RPA class	OS after first recurrence (week)	AEs¶
1	70	Male	20	M	PR	7	73.1	2
2	66	Male	50	U	SD	7	28.6	1
3	64	Female	30	M	SD	7	28.4	1
4	75	Male	40	U	SD	7	93.1	1
5	55	Male	50	U	SD	7	33.0	1
6	77	Male	50	M	SD	7	46.7	2
7	60	Female	50	M	SD	7	44.9	1
Median	66	Male 5 Female 2	50	M 4 U 3	PR 1 SD 6	7	44.9	
Historical control	72 (median)	Male 8 Female 7	40	M 8 U 6 ND 1	SD 3 PD 12	7 (median)	17.3 (median)	

Abbreviations: AE, adverse event; KPS, Karnofsky Performance Status; MGMT, O^6^-methylguanine-DNA-methyltransferase; PD, progressive disease; PFS, progression-free survival; PR, partial response; OS, overall survival; RPA, recursive partitioning analysis; SD, stable disease.

*MGMT promoter status; ND, not detectable; M, methylated; U, unmethylated.

†Response was assessed according to Macdonald criteria.

¶AE score was based on Common Terminology Criteria for Adverse Events (CTCAE) version 4.0.

## DISCUSSION

In this study, we first demonstrated the independent prognostic relevance of tumor-GSK3β activity as detected by the increased level of pGSK3β^Y216^ that inversely associated with survival of GBM patients, while no correlation was found between the expression level of mRNA and total protein and the prognosis (data not shown). This suggests aberrant GSK3β activity in the tumor cells as a new prognostic factor in GBM and clinically rationalizes the strategy of targeting of this kinase for treatment of GBM. We also found therapeutic effects of the existent GSK3β-inhibiting medicines against GBM in the experimental and preclinical settings as well as a clinical study for the chemotherapy-resistant refractory patients. Previously, we and others have reported that inhibition of GSK3β activity attenuated proliferation and invasion of GBM cells and sensitized them to chemotherapeutic agents and radiation by modulating distinctive molecular pathways [19, 29]. Consistent with our earlier study [29], we confirmed that inactivation of GSK3β suppressed the molecular axis involving FAK and Rac1 in the present preclinical study. It is notable that the cocktail of GSK3β-inhibiting medicines tested in this study attenuated survival, migration and invasion of GBM cells and animal-model tumors. This cocktail also revitalized TMZ against recurrent GBMs and this effect was associated with decreased MGMT expression in the patient, thereby resulting in improvement of prognosis in patients of GSK high group in spite of low EOR. Our present study reinforces the cancer therapeutic effect of GSK3β inhibition by repurposing these medicines for GBM. It also suggests the necessity for the development and approval of specific GSK3β inhibitors to achieve efficient therapeutic effect for diseases including cancer.

Combination of multiple drugs with different mechanisms of action and/or different binding sites of a same target is often required for cancer chemotherapy to optimize the therapeutic effects, minimize the adverse effects, and prevent the development of treatment resistance [32, 33]. CLOVA cocktail showed additive effect on GBM proliferation and invasion (Figures 2 and 3; Supplementary Figures 3 and 4) but no adverse effect in the treated mice. Notably, cell viability showed no difference between control group and the group treated with CLOVA cocktail at the point of 8 hr when invasion assay was evaluated (data not shown). Nowicki et al also demonstrated that the effect of GSK3β inhibition on migration occurred earlier time points than effects on cell viability [34]. There are several ways of inhibiting GSK3β activity. They include up-regulation of pGSK3β^S9^ (inactive form), down-regulation of pGSK3β^Y216^ (active form) and targeting the catalytic domain and/or the adenosine triphosphate (ATP)-binding pocket structure in this enzyme (Supplementary Figure 7) [9]. Cimetidine potently blocks the catalytic domain of GSK3β. Lithium has dual inhibitory effects on GSK3β to induce phosphorylation of serine 9 (S9) residue and to compete with Mg^2+^ that is required for GSK3β-dependent substrate phosphorylation. Olanzapine also increases the level of S9 phosphorylation and docks with the ATP-binding pocket of GSK3β. Valproate directly inhibits the activated GSK3β wherein Y216 residue is phosphorylated. Aberrant regulation of pGSK3β^Y216^ could promote the malignant features of GBM [18, 19, 35] since it is an independent prognostic factor for GBM patient in the present study. Elevated pGSK3β^Y216^ fraction contributed the malignant feature of pancreatic cancer [36] and inhibition of pGSK3β^Y216^ enhanced the effect of imatinib inducing apoptosis in chronic myeloid leukemia [35]. These knowledge and observations support the present study showing that lithium and valproate inhibiting pGSK3β^Y216^ exhibited stronger antitumoral effect than cimetidine and olanzapine that induce pGSK3β^S9^.

We define or regard the CLOVA cocktail as a single drug (the mixture of four constituents) basically in this study, and showed its effects against GBM cells proliferation and invasion. This effect is consistent with a substantial number of previous studies including ours showing the tumor-promoting roles of GSK3β, therapeutic effect of its inhibition and the underlying biological mechanisms in various cancer types including GBM [9–16]. The less effect of cimetidine and olanzapine (both repurposed, but not specific GSK3β inhibitors) on GBM cell proliferation at their limited dose ranges does not always make a denial of the solid evidence for the critical role of GSK3β in promoting proliferation of tumor cells including GBM.

Each of the GSK3β-inhibiting medicines has unique cancer therapeutic mechanisms other than its GSK3β-inhibitory effect. Cimetidine is a histamine H_2_ receptor antagonist prescribed for gastroduodenal ulcers and its anti-tumor activity was reported in many cancer types including glioma. It was shown to attenuate cell proliferation and migration by blocking histamine that promotes tumor angiogenesis [37, 38]. Lithium is a classical, non-competent GSK3β inhibitor [23] and prescribed for the treatment and prophylaxis of bipolar mood disorders and depression [39]. A previous study showed a reversible dose-dependent effect of lithium on attenuation of glioma cell invasion via inhibition of GSK3β activity [34]. Olanzapine is one of the most frequently prescribed anti-psychotic agents for schizophrenia and bipolar disorder [40]. It has attracted the attention of neuro-oncologists because of its ability to enhance the cytotoxic effects of TMZ on GBM [40]. GSK3β inhibition by olanzapine stimulated adenosine monophosphate (AMP)-activated protein kinase catabolic action followed by induction of p53-dependent autophagy, which enhances the pro-apoptotic effect of TMZ [41]. Valproate is approved for treatment of epileptic seizure, bipolar disorder, and migraine. It acts as a histone deacetylase inhibitor [42] that alters the chromatin structure, consequently increasing DNA accessibility to anticancer drugs and enhancing the effect of radiation [43, 44], in addition to other anticancer mechanisms [45]. In addition to their effects on GSK3β, lithium and valproate induced autophagy via inhibition of inositol monophosphatase [46] and modulation of oxidative stress [47], respectively, resulting in cancer therapeutic effect. Anti-tumor effects of valproate were tested by a number of clinical trials [48] and some studies showed significant survival in GBM patients treated with valproate [43, 45, 47]. Recently, a meta-analysis invalidated the use of VPA for reasons other than seizure control in patients with newly diagnosed GBM outside clinical trials [50], suggesting the limitations of single medication with GSK3β-inhibitory drug.

Based on the results of our experimental and preclinical studies shown here, we conducted a clinical study for the TMZ-resistant recurrent GBM patients by repositioning of the GSKβ-inhibiting medicines in combination with TMZ (UMIN:00005111). Patients treated with this regimen showed significant longer survival (median survival 11.2 months after recurrence) than historical control (median survival 4.4 months) treated with TMZ alone even though all the former patients were classified as RPA 7 in which the bad prognosis is predicted [31]. Immunohistochemical analysis of the patients’ tumors (Figure 4) suggested that the repurposed drugs inhibited GSK3β activity in the tumor cells, decreased expression of MIB-1 cell proliferation marker, and reduced invasion by the residual tumor cells. Although this clinical study has limitations such as single institution and small number of patients enrolled, it was first to provide the evidence of GSK3β-targeted cancer therapy based on drug repositioning. Not all, but combination of some GSK3β-inhibiting medicines might be enough for inhibition of aberrant GSK3β in the tumors. The next step toward clinical translation of this therapeutic strategy is to plan a large-scale, prospective multicenter trial to establish the optimum combination of the GSK3β-inhibiting medicines. A subsequent trial is also important to compare the effects of them in combination with TMZ and with bevacizumab. It will clarify whether the GSK3β-targeted therapy can prevent the GBM invasion enhanced in the bevacizumab-resistant tumors [51, 52].

Accumulating evidence has shown that inhibition of GSK3β provides dual benefits for the treatment of GBM patients by attenuation of tumor progression [17, 29] and protection from neurodegenerative effects of irradiation [53, 54]. This study also supports and promotes future clinical translation of the GSK3β-targeted therapy by drug repositioning. To ascertain the effect of GSK3β-targeted therapy with repurposing drugs, large multicenter clinical trial is currently in progress to validate the efficacy and safety of CLOVA cocktail in combination with TMZ, and best combination of the drugs composed of the cocktail in patients with recurrent GBM.

## MATERIALS AND METHODS

### Established predictive factors and GSK3β activity in the tumor of GBM patients

We examined 57 patients with GBM (Supplementary Table 1) for influence of tumor-GSK3β activity on their survival. This study was approved by Kanazawa University Medical Ethics Committee. Fresh tumor tissues were obtained by biopsy and surgery and the remaining tissue samples were fixed in 4% paraformaldehyde and embedded in paraffin for routine histopathologic examination and immunohistochemical analysis. The histological diagnosis was determined according to the revised World Health Organization criteria [55].

Established predictive factors including age [56], Karnofsky Performance Status (KPS) [56], EOR [57], and methylation in O^6^-methylguanine-DNA methyltransferase (MGMT) gene promoter [58] were analyzed in these patients. Progression-free survival (PFS) and overall survival (OS) were calculated from the initial operation to last magnetic resonance imaging (MRI) and to death, respectively. Log-rank test for univariate analysis was performed to determine statistical significance of Kaplan-Meier survival curve. Cox proportional hazard regression model was used to identify the multivariate predictors of survival.

Methylation of MGMT promoter was examined by methylation-specific PCR. Genomic DNA was extracted from paraffin sections of tumor by using QIAamp DNA FFPE Tissue kit^™^ (QIAGEN, Hilden, Germany). Sodium bisulfite conversion of 1 μg DNA was performed using an EpiTect Bisulfite Kit (QIAGEN) according to the manufacture's protocol. Methylation-specific PCR of bisulfite-converted DNA was carried out by a nested, two-stage PCR approach as described previously [59] using GeneAmp PCR System 2700 (Applied Biosystems, Foster city, CA, USA). U87 and U138 cell lines were used as methylated and unmethylated controls, respectively. Amplified PCR products were separated by 3% agarose gel electrophoresis and visualized with ethidium bromide.

Tumor GSK3β activity was examined immunohistochemically for the level of phosphorylation at tyrosine (Y) 216 residue (pGSK3β^Y216^). Representative paraffin sections of the tumors were immunostained with GSK3β and pGSK3β^Y216^ antibodies (BD Biosciences, San Jose, CA, USA) (Supplementary Table 2), using Envision+ Kit (DAKO Japan, Kyoto, Japan) as described previously [60]. Images were acquired with a BZ-X700 microscope (Keyence, Osaka, Japan) and digitally processed with the Keyence Analysis Software. The patients were classified into 2 groups depending on the level of pGSK3β^Y216^ as “GSK high” (≥ 50% of the tumor cells were labeled) and “GSK low” (< 50% of the tumor cells were labeled). Immunohistochemical evaluation was carried out by neuropathologist (H.S.) and oncologist (T.M.) in independent readings.

### Cell culture

Human glioma cell lines T98, U87, U251, and U138 were obtained from American Type Culture Collection (ATCC) in 2009. These cell lines were characterized in the resource institute by short tandem repeat profile analysis. Authentication of the cell lines was unnecessary because cells were expanded by culturing them for less than two passages and stored at −80°C. Low-passage cells were used for experiments within the period of 6 months after resuscitation. They were maintained in Dulbecco's modified Eagle medium (DMEM) supplemented with 10% fetal bovine serum (FBS) at 37°C with 5% CO_2_. All cells were *Mycoplasma* free.

### Existent GSK3β-inhibiting medicines and temozolomide

Six GSK3β-inhibitory drugs were reported; lithium [61, 62], cimetidine [63], gemifloxacin [63], hydroxychloroquine [63], olanzapine [64], and valproate [24, 65]. Of these, 4 drugs were selected because of their general usage for the symptoms of the patients suffering from brain tumor; cimetidine for gastroduodenal ulcer, lithium and olanzapine for mood disorder, and valproate for seizure.

imetidine, lithium, olanzapine and valproate were purchased from WAKO Pure Chemicals (Osaka, Japan). For stock solutions, cimetidine and olanzapine were dissolved in dimethyl sulfoxide (DMSO), and lithium and valproate were dissolved in distilled water. As described in the Results, the concentrations of these drugs used in culture medium were as follows: cimetidine: 0.1, 0.5, and 1 mM; lithium: 1, 5, and 10 mM; olanzapine: 0.1, 0.5, and 1.0 μM; and valproate: 1, 5, and 10 mM. The combination of these drugs at their lowest concentration was referred as “CLOVA cocktail” by taking the initial letters of each drug.

Temozolomide (TMZ) was purchased from Sigma-Aldrich (St Louis, MO, USA). The concentration of this agent was 200 μM for cell culture and 100 mg/kg for animal model experiment.

### Western blot analysis

Cellular protein was extracted from cultured cells following the treatment using lysis buffer (Sigma-Aldrich) containing a mixture of protease and phosphatase inhibitors (Sigma-Aldrich). A 15-μg aliquot of whole protein extract was analyzed by Western immunoblot for the protein of interest (Supplementary Table 2), as described previously [66]. β-actin was used as loading control. Immunoblot signals were measured using the CS analyzer (version 2.0; ATTO, Tokyo, Japan).

### Cell invasion assay

Cell invasion assays were performed using modified Boyden chambers consisting of Transwell with pre-coated Matrigel membrane filter inserts in 24-well tissue culture plates (BD Biosciences) as described previously [66]. Serum-deprived cells suspended in DMEM containing 0.1% fetal bovine serum (FBS) were added to each Transwell. After incubation at 37°C for 8 h, non-invading cells were removed by wiping the upper side of the membrane, and the invading cells were fixed with methanol and stained with Diff-Quick Kit (Sysmex, Kobe, Japan). The invading cells on the filter were counted from 8 randomly selected high-power microscopic fields. The mean number of cells and standard deviations were calculated.

### Cell proliferation assay

Alamar Blue assay (Biosource, Camarillo, CA, USA) was performed according to manufacturer's manual. Practically, 1,000 cells of each population were seeded in wells of 96-well plastic plates in 200 μl of culture medium supplemented with 0.1% FBS. The plates were incubated for 4 h at 37°C, and 20 μl Alamar Blue (10% of total volume) was added to the cells and incubated. The plate was read on a fluorescence plate reader (excitation, 30 nm; emission, 590 nm) at 0, 24, 48, 72, and 96 h. Averages of the fluorescence values were calculated and plotted. To investigate the influence of drugs on cell proliferation, cells were serum starved for 24 h and seeded in the proliferation assay format. The cells were then treated with various concentrations of each drug, CLOVA cocktail, TMZ, or with the latter two in combination.

### Mouse model of GBM and treatment

Following an institutional review board-approved protocol, we generated a mouse brain tumor model of human GBM by retrovirus-mediated introduction of the mutant K-ras gene (K-ras^G12V^) in neurospheres derived from the brain of p16^Ink4A−/−^/p19^Arf−/−^ mouse and transplantation of them into the brain of wild-type mice according to our previous study [67]. The activation of GSK3β was validated both in the cultured sphere and the *in vivo* developed tumor by Western blot (data not shown). These mice were treated with the CLOVA cocktail or TMZ either alone or in combination. Practically, the 20 mice were randomly assigned to four groups for treatment with H_2_O/DMSO as control (*n* = 5), CLOVA cocktail alone (*n* = 5), TMZ alone (*n* = 5), and CLOVA cocktail and TMZ in combination (*n* = 5). Each drug of CLOVA cocktail at dose equivalent to the human dose was normalized to mice weights as follows; cimetidine 13.3 mg/kg; lithium 6.7 mg/kg; olanzapine 167 μg/kg; and valproate 13.3 mg/kg. CLOVA cocktail was orally administered every day and TMZ was administered intraperitoneally at 100 mg/kg for first 5 days. Following 2 weeks of treatment, all 20 mice were euthanized. For survival study, the 10 mice were treated with H_2_O/DMSO (*n* = 5) or CLOVA cocktail (*n* = 5). All animal experiments followed the Guidelines for the Care and Use of Laboratory Animals at Kanazawa University that covers the national guideline.

Representative paraffin sections of the tumors were immunostained for glycogen synthase phosphorylated at S641 residue (pGS^S641^), MIB-1, nestin, and focal adhesion kinase phosphorylated at Y397 and Y861 residue (pFAK^Y397^ and pFAK^Y861^) using the respective antibodies (Supplementary Table 2) as described above. Nestin-positive tumor cell clusters were scored to evaluate the degree of invasion as described previously [68]. Representative paraffin sections of the tumors were immunostained with a 1:50 dilution of anti-active Rac1 monoclonal antibody (NewEast Biosciences, King of Prussia, PA, USA) and then incubated in biotinylated horse anti-mouse IgG antibody (1:50; Vector Laboratories, Burlingame, CA, USA), followed by incubation with Alexa Fluor 594-labelled streptavidin (1:500; Vector Laboratories). Non-immune mouse serum at 1:50 dilution was used as negative control in immunostaining. The stained sections were mounted with the mounting medium for fluorescence with 4′,6′-diamidino-2-phenylindole (DAPI; Santa Cruz Biotechnology, Heidelberg, Germany). Images were captured with a BZ-X700 microscope (Keyence).

### Design of clinical study

The single center, single-arm, clinical study investigated the efficacy and safety of CLOVA cocktail in combination with TMZ in patients with recurrent GBM in Kanazawa University Hospital from January 2009 to October 2010 under approval by Medical Ethics Committee of Kanazawa University (UMIN Clinical Trial Registry: UMIN000005111). Written informed consent was obtained from all patients. Primary endpoint was OS after first recurrence. Secondary endpoint was safety compared with matched historical control population treated with TMZ alone.

#### Patients

Adult patients (age ≥ 18 years) with histologically proven recurrent GBM after standard chemoradiation therapy with TMZ were eligible for the study (Table 2).

#### Treatment

CLOVA cocktail comprising 800 mg cimetidine, 400 mg lithium, 10 mg olanzapine, and 800 mg valproate was orally administered daily. Dose of each drug was determined based on medical package insert, and therapeutic drug monitoring of lithium and valproate was performed every month. Maintenance TMZ at 200 mg/m^2^/day was administered for 5 consecutive days every 4 weeks (Figure 5A).

#### Efficacy and safety assessments

A baseline gadolinium-enhanced MRI scan was performed within 1 week before beginning the treatment. MRI was repeated every 1 month. Progression was based on investigators’ clinical and radiological assessment according to Macdonald criteria [30]. Safety was evaluated by descriptively summarizing adverse events (AEs), laboratory assessments, and physical examinations. All AEs were recorded according to Common Terminology Criteria for Adverse Events (CTCAE) version 4.0.

### Matched historical controls

Matched historical controls were identified from records of patients with recurrent GBM who had received treatment with maintenance TMZ at 200 mg/m^2^/day in Kanazawa University Hospital (Supplementary Table 3) same as the patients enrolled the study.

### Statistical analyses

Statistical significance was determined using Student's *t*-test, Mann-Whitney *U* test and Fisher's exact test for comparison of two groups as appropriate. Log-rank analysis was used to determine statistical significance of Kaplan-Meier survival curve. All analyses were performed using SPSS statistical package version 19 for Macintosh (IBM Japan Ltd., Tokyo, Japan). The significance level was set at *p* = 0.05.

## SUPPLEMENTARY MATERIALS FIGURES AND TABLES


