# Supplementary material for: Biological basis and clinical study of glycogen synthase kinase- 3β-targeted therapy by drug repositioning for glioblastoma
Source: Oncotarget. 2017 Feb 9;8(14):22811–24. doi: 10.18632/oncotarget.15206 (PMC5410264; doi:10.18632/oncotarget.15206)
Supplement: Supplementary file 1 [file oncotarget-08-22811-s001.pdf]

# Biological basis and clinical study of glycogen synthase kinase-3β-targeted therapy by drug repositioning for glioblastoma

## Supplementary Materials

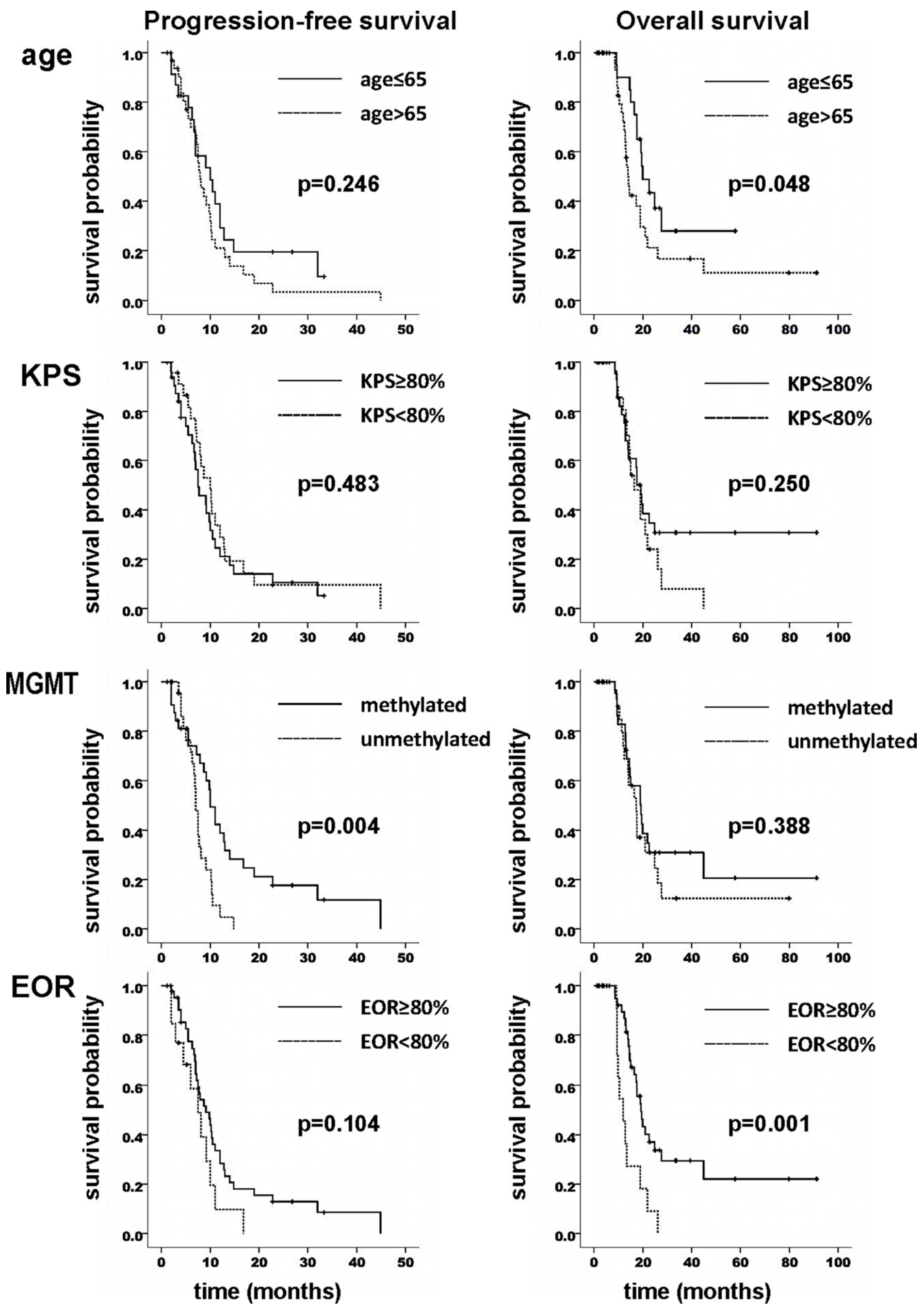


**Supplementary Figure 1: Comparison of progression-free and overall survivals of 57 patients with glioblastoma (Supplementary Table 1) by Kaplan-Meier method according to the respective approved prognostic factors; patient's age, Karnofsky Performance Scale (KPS), methylation of O6-methylguranine-DNA-methyltransferase (MGMT) gene, and extent of resection (EOR).**

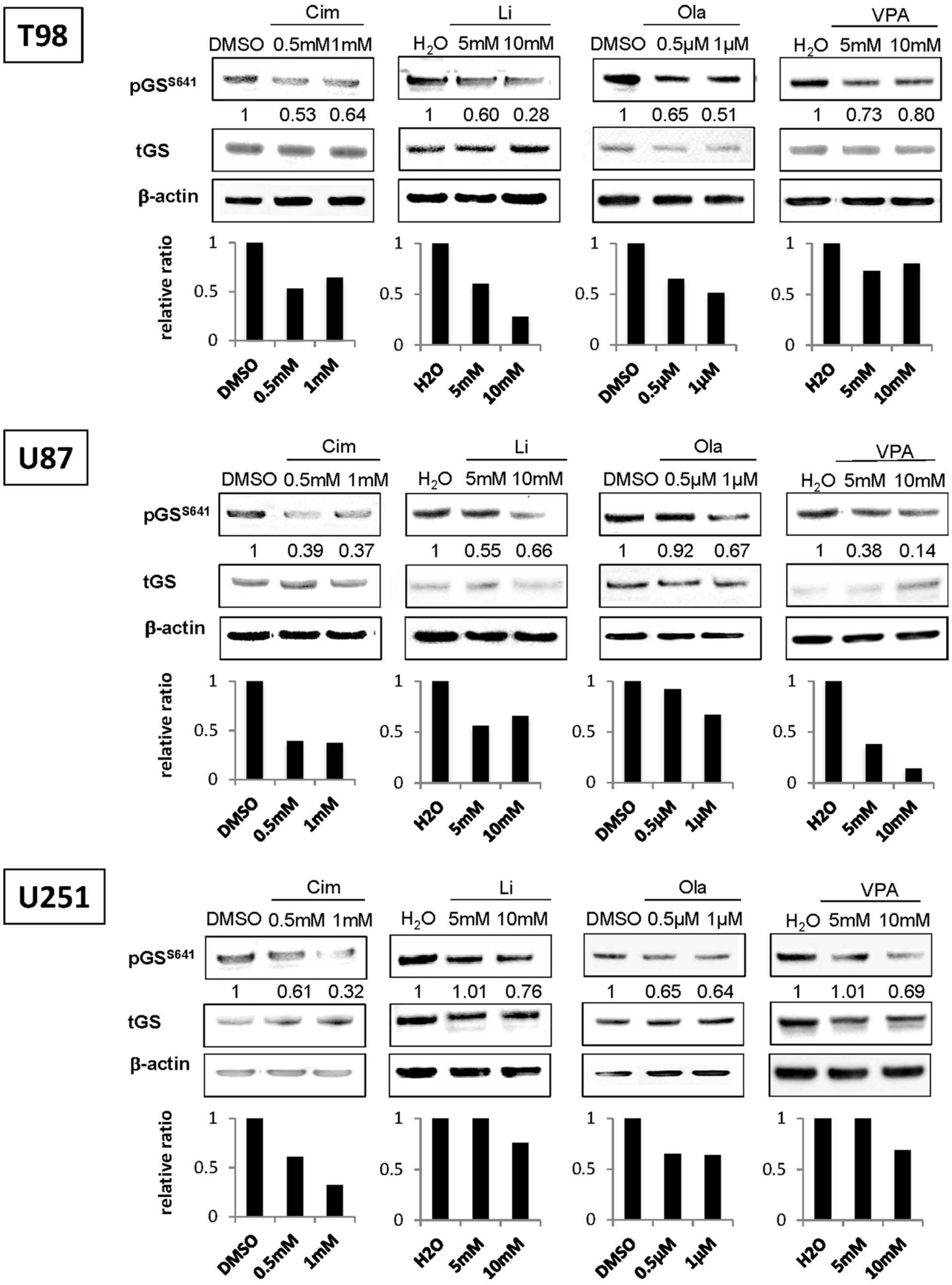


**Supplementary Figure 2: GSK3β-inhibitory effect of four existing drugs.** All drugs inhibited the activity of GSK3β estimated by the level of $pGS^{S641}$. The value below each lane shows the relative level of $pGS^{S641}$ quantified by densitometry and normalized to that of total (t) GS. All blots were cropped for clarity and conciseness of the images.

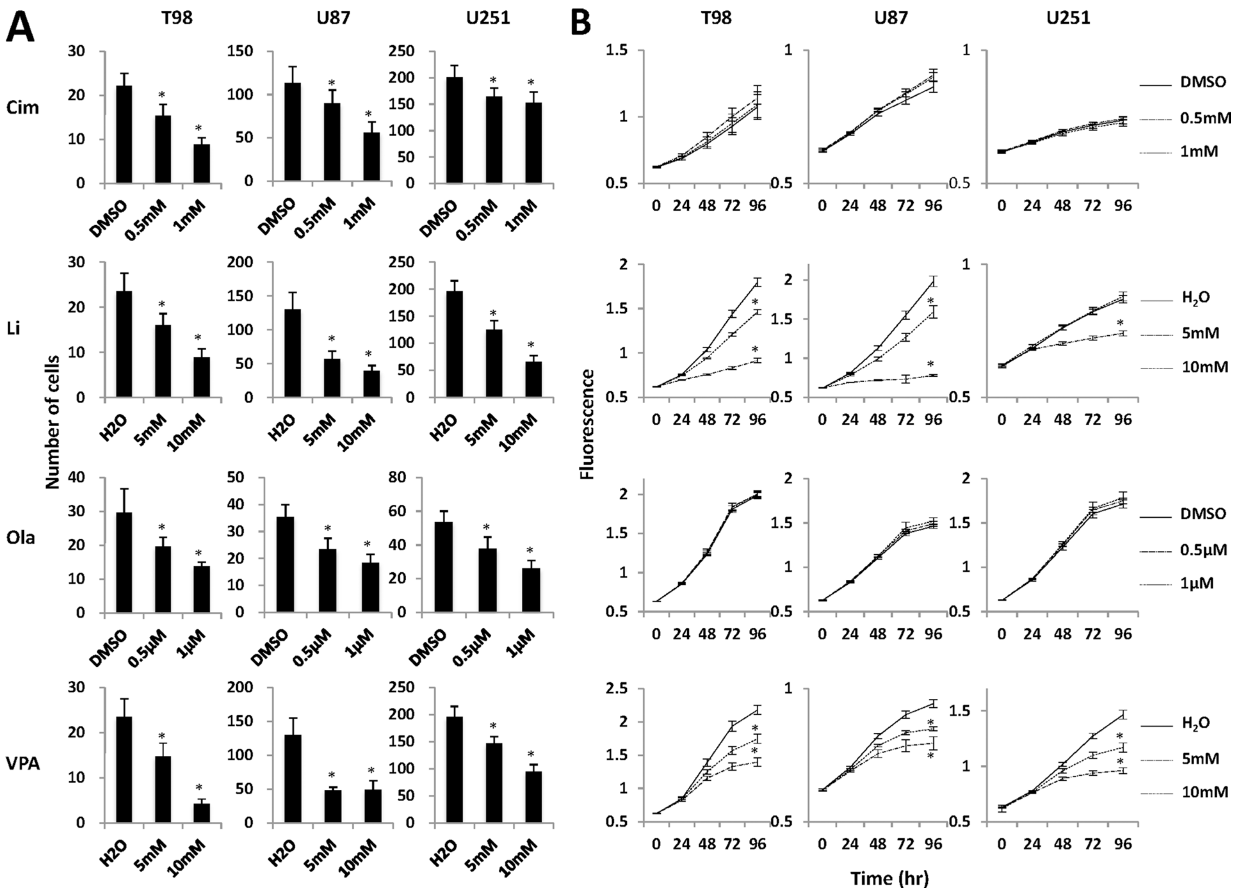


**Supplementary Figure 3: Effect of GSK3β-inhibitory drugs on the glioblastoma cell invasion and proliferation.** (**A**) Invading cells through a Matrigel-coated Transwell chamber were scored for cells treated with or without each drug at the indicated concentration for 8 hours. The mean number of cells in eight high-power microscopic fields was calculated with SDs. (**B**) Growth curve analysis of glioblastoma cell lines treated with or without each drug at the indicated concentration for 24 to 96 hrs. The plate was read on absorption plate reader at the indicated time points. A and B: $*p < 0.05$, statistically significant difference between cells treated with DMSO/H2O and each drug. Cim: cimetidine; Li, lithium; Ola, olanzapine; VPA, valproic acid.

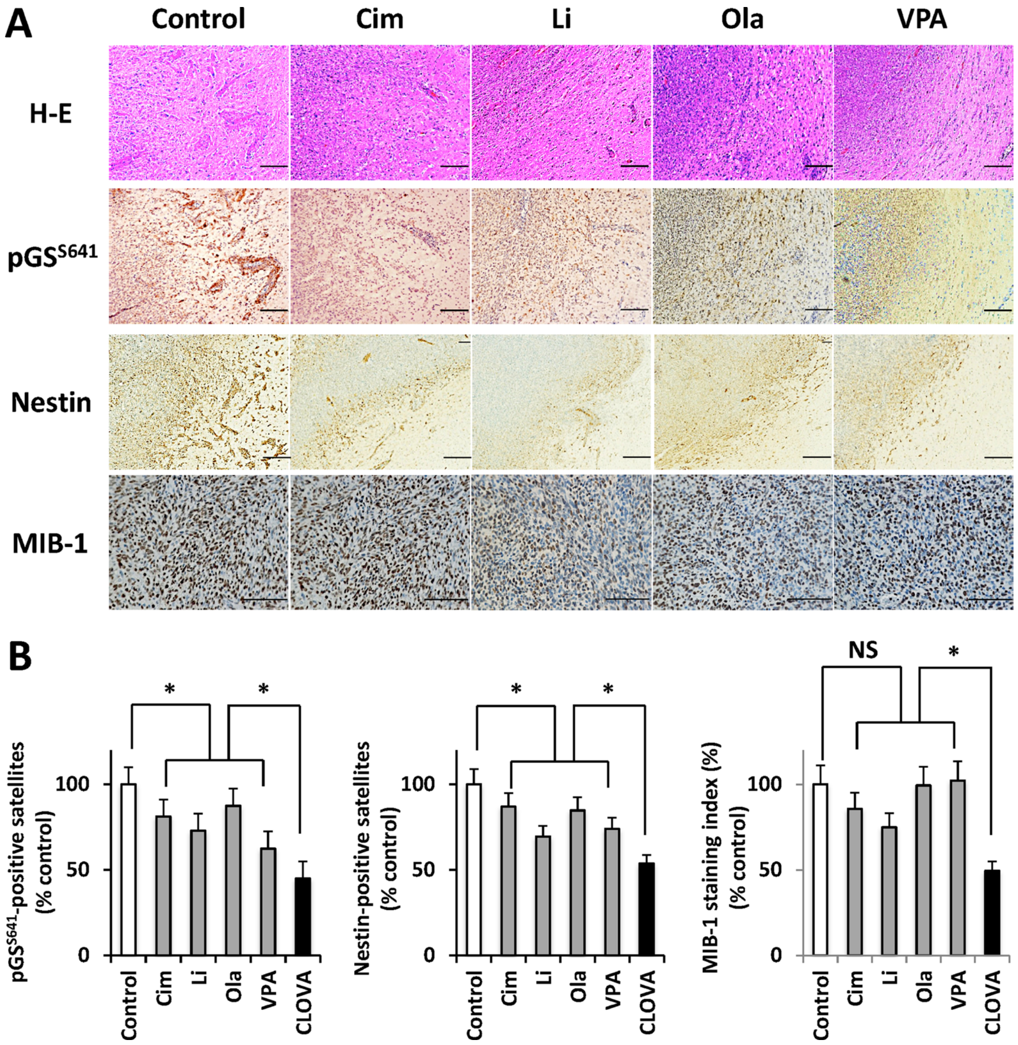


**Supplementary Figure 4:** (**A**) Representative histopathological and immunohistochemical findings of the serial sections of mouse brain tumors stained with H.E. and immunostained for pGSS641, nestin and MIB-1, respectively. Mice treated with each drug showed well-demarcated border between tumor and normal brain tissue compared to those treated with the control reagents. The levels of pGSS641 and nestin was decreased in invading cell clusters. MIB-1 staining index was almost equal between mice as control and those treated. Scale bar, 100 µm. (**B**) Comparison of the effects of each GSK3b-inhibiting medicine and the CLOVA cocktail on the number of pGSS641- and nestin-positive cell clusters and on MIB-1 stainng index. In the respective assays, numbers of pGSS641- and nestin-positive cell clusters and MIB-1-positive/total number of tumor cells per high-power microscopic field were scored for the tumors in mice untreated (control) and treated with the respective reagents. The mean number of the clusters or the index in 3 fields was calculated with standerd deviations. *$p < 0.05$. Cim: cimetidine; Li, lithium; Ola, olanzapine; VPA, valproic acid.

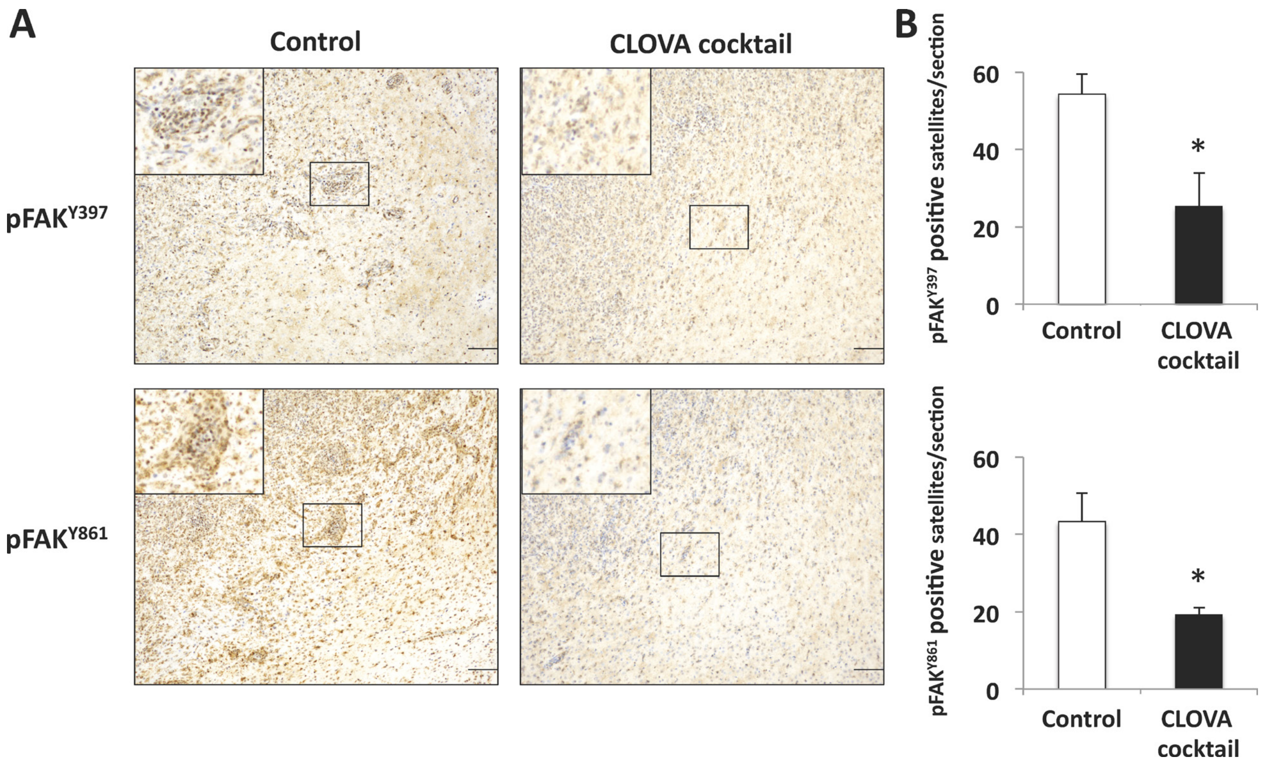


**Supplementary Figure 5:** (**A**) Representative immunohistochemical findings of brain tumors for phosphorylation of FAK (pFAKY397 and pFAKY861) in untreated (control) and CLOVA cocktail-treated mice. Phosphorylation of both tyrosine (Y) residues in FAK were decreased in the satellite lesions of tumor in mice treated with CLOVA cocktail. The magnified image of the area in the square is shown in the left upper corner of each panel. Scale bars, 100 µm. (**B**) Effects of CLOVA cocktail on the number of pFAKY397- and pFAKY861-positive cell clusters in the brain tumors. *$p < 0.05$.

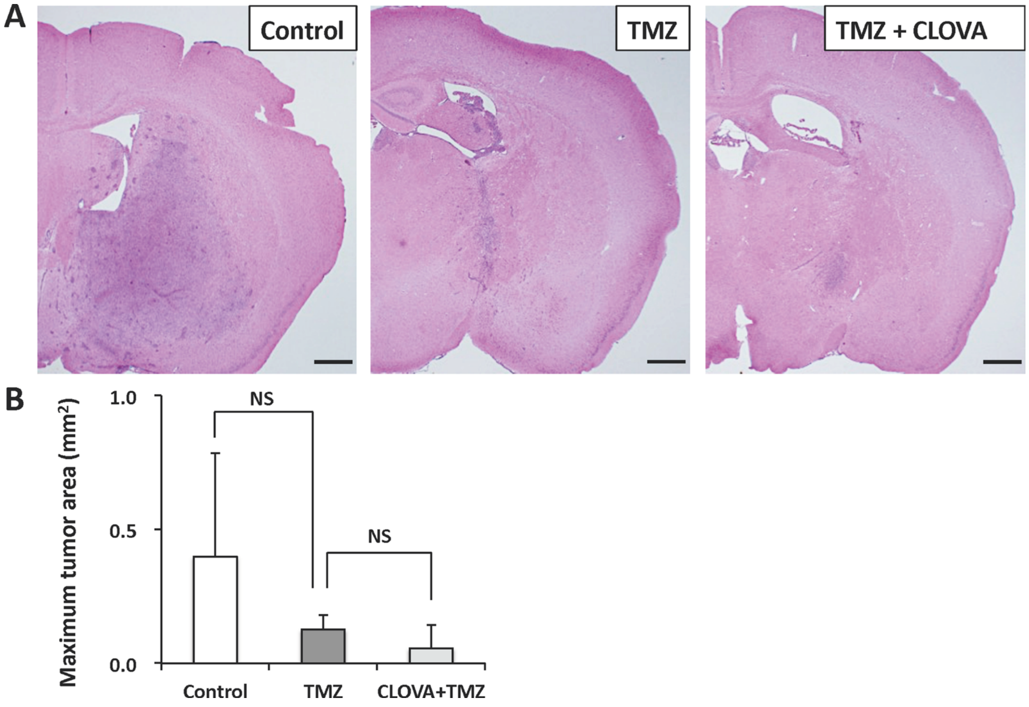


**Supplementary Figure 6:** (**A**) Representaive H.E.-stained cut sections of the brains of mice untreated (control) and treated with TMZ alone and in combination with the CLOVA cocktail. (**B**) Effect of CLOVA cocktail alone and in combination with TMZ on the maximum tumor area ($mm^2$; mean ± SD) of mouse brain tumors. *Scale bars*, 500 µm. NS, not statistically significant; TMZ, temozolomide.

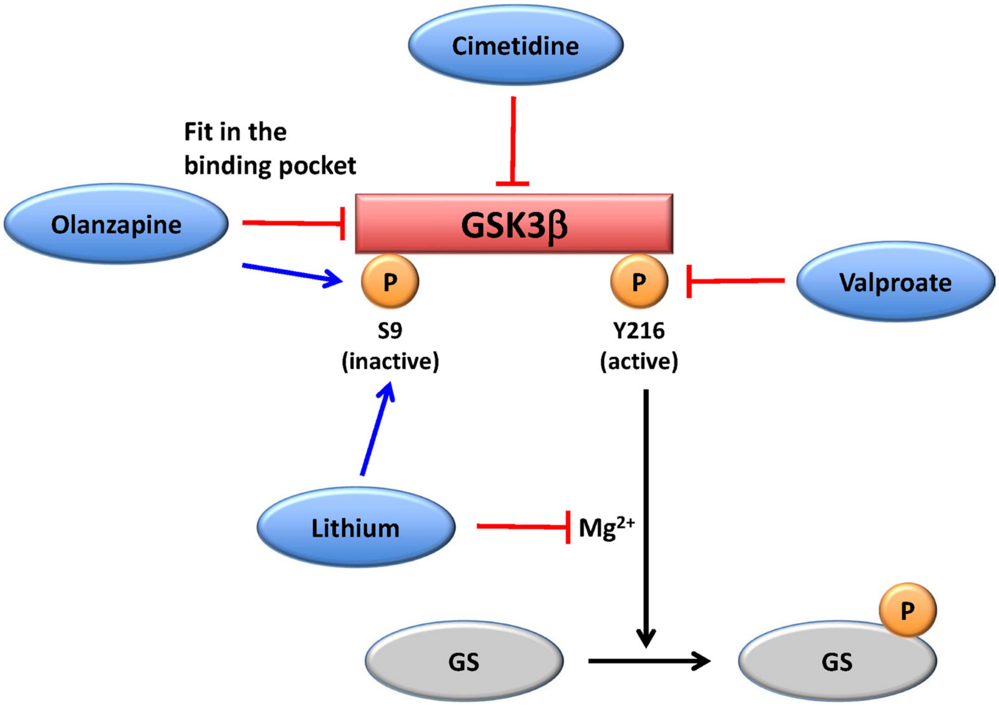


**Supplementary Figure 7: Schematic representation of the multiple actionable targets of each GSK3β-inhibitory drug in the molecule of GSK3β (cited from Ref.** [9] with modification). Cimetidine has at least two distinct binding modes accessible to the ligands within the GSK3β binding pocket. Lithium inhibits GSK3β activity by competing with $Mg^{2+}$ that is required for GSK3β-dependent substrate phosphorylation. Lithium and olanzapine induce the phosphorylation of the serine (S) 9 residue. Olanzapine also docks with the adenosine triphosphate-binding pocket of GSK3β. Valproate directly inhibits activated GSK3β in which tyrosine (Y) 216 residue is phosphorylated. Circled P, phosphorylation; GS, glycogen synthase; GSK3, glycogen synthase kinase 3β

**Supplementary Table 1: Characteristics of the patients with low expression of pGSK3β$^{Y216}$ (GSK low) and high expression of pGSK3β$^{Y216}$ (GSK high)**

| | Total (*n* = 57) | GSK low (*n* = 22) | GSK high (*n* = 35) | *p** |
|---|---|---|---|---|
| Age ≥ 65 (%) | 32 (56.1) | 13 (59.1) | 19 (54.3) | 0.72 |
| Female (%) | 21 (36.8) | 8 (36.3) | 13 (37.1) | 0.95 |
| KPS ≥ 80 (%) | 32 (56.1) | 10 (45.5) | 22 (62.9) | 0.19 |
| Methylated MGMT promoter (%) | 34 (59.6) | 15 (68.2) | 19 (54.3) | 0.29 |
| Extent of resection ≥ 80% (%) | 44 (77.2) | 19 (86.4) | 26 (74.3) | 0.28 |
| Tumor location | | | | |
| frontal | 21 (36.8) | 10 (45.5) | 11 (31.4) | 0.29 |
| temporal | 15 (26.3) | 3 (13.6) | 12 (32.3) | 0.08 |
| parietal | 8 (14.0) | 5 (22.7) | 3 (8.6) | 0.13 |
| occipital | 4 (7.0) | 2 (9.1) | 2 (5.7) | 0.65 |
| cerebellum | 2 (3.5) | 0 (0.0) | 2 (5.7) | 0.25 |
| thalamus | 1 (1.8) | 1 (4.5) | 0 (0.0) | 0.20 |
| brain stem | 1 (1.8) | 0 (0.0) | 1 (2.9) | 0.42 |
| multicentric | 5 (8.8) | 1 (4.5) | 4 (11.4) | 0.37 |

Abbreviations: GSK3β, glycogen synthase kinase 3β; KPS, Karnofsky Performance Scale; MGMT, O$^6$-methylguranine-DNA-methyltransferase.
*Fisher's exact test.

**Supplementary Table 2: Source and working dilutions of the primary antibodies used for Western blotting, immunohistochemical and immunofluorescence staining**

| Antibody | MW (kD) | Source | Working dilution | Company |
|---|---|---|---|---|
| pGS$^{S641}$ | 90 | Rabbit | 1:1,000 (WB), 1:400 (IHC) | Cell Signaling |
| GS | 90 | Rabbit | 1:1,000 (WB), 1:400 (IHC) | Cell Signaling |
| β-actin | 42 | Mouse | 1:2,000 (WB) | WAKO |
| pGSK3b$^{Y216}$ | 46 | Mouse | 1:400 (IHC) | BD Biosciences |
| GSK3β | 46 | Mouse | 1:400 (IHC) | BD Biosciences |
| nestin | 260 | Mouse | 1:400 (IHC) | BD Biosciences |
| MIB-1 | 359 | Rabbit | 1:800 (IHC) | Thermo Scientific |
| MGMT | 21 | Rabbit | 1:400 (IHC) | Cell signaling |
| pFAK$^{Y397}$ | 119 | Rabbit | 1:200 (IF) | Abcam |
| pFAK$^{Y861}$ | 119 | Rabbit | 1:200 (IF) | Abcam |
| Active Rac1 | 21 | Mouse | 1:50 (IF) | NewEast Biosciences |

Abbreviations: FAK, focal adhesion kinase; GS, glycogen synthase; GSK3β, glycogen synthase kinase 3β; IF, immunofluorescence; IHC, immunohistochemistry; MGMT, O$^6$-methylguanine-DNA methyltransferase; MW, molecular weight; pFAK$^{Y397}$ or pFAK$^{Y861}$, FAK phosphorylated at tyrosine (Y) 397 or 861 residue; pGS$^{S641}$, GS phosphorylated at serine (S) 641 residue; pGSK3β$^{Y216}$, GSK3β phosphorylated at Y216 residue; WB, Western blotting.

**Supplementary Table 3: Ccharacteristics of the patients with glioblastoma in historical control group**

| Case | Age | Sex | KPS | MGMT* | Response† | RPA class | OS after first recurrence (week) |
|---|---|---|---|---|---|---|---|
| 1 | 70 | Female | 40 | U | PD | 7 | 5.9 |
| 2 | 75 | Male | 50 | M | PD | 6 | 29.9 |
| 3 | 69 | Female | 30 | M | PD | 6 | 12.1 |
| 4 | 79 | Female | 30 | M | PD | 6 | 6.0 |
| 5 | 49 | Male | 60 | ND | SD | 5 | 35.3 |
| 6 | 64 | Male | 50 | M | SD | 6 | 36.0 |
| 7 | 72 | Female | 40 | U | PD | 6 | 12.0 |
| 8 | 51 | Female | 30 | M | PD | 7 | 16.3 |
| 9 | 81 | Male | 30 | U | PD | 7 | 29.7 |
| 10 | 79 | Male | 30 | M | PD | 7 | 5.9 |
| 11 | 54 | Male | 30 | M | PD | 7 | 17.7 |
| 12 | 81 | Female | 40 | M | PD | 7 | 27.3 |
| 13 | 86 | Male | 40 | U | SD | 7 | 34.0 |
| 14 | 78 | Female | 40 | U | PD | 7 | 11.6 |
| 15 | 63 | Male | 40 | U | PD | 7 | 17.3 |
| Median | 72 | Male 8<br>Female 7 | 40 | M 8<br>U 6<br>ND 1 | SD 3<br>PD 12 | 7 | 17.3 |

Abbreviations: KPS, Karnofsky Performance Status; MGMT, $O^6$-methylguanine-DNA-methyltransferase; PD, progressive disease; PFS, progression-free survival; PR, partial response; OS, overall survival; RPA, recursive partitioning analysis; SD, stable disease.

*MGMT promoter status; ND, not detectable; M, methylated; U, unmethylated.

†Response was assessed according to Macdonald criteria.
